# Our Duty in Regard to the World’s Columbian Dental Congress

**Published:** 1893-06

**Authors:** C. N. Peirce


### Domestic Correspondence.

### OUR DUTY IN REGARD TO THE WORLD'S COLUMBIAN DENTAL CONGRESS.

To the Editor:

Sir,—The World's Columbian Dental Congress is at the present time eliciting much interest. Is it possible that misgivings regarding its success are entertained? The query must come home to every one in the dental profession as to whether he or she can afford to have a feeling of uncertainty about a matter which so deeply concerns the dentists of this country. It is at this moment a matter of not the slightest consequence, and one which we cannot now stop to consider, whether those in authority or holding positions of importance or trust were of our own choosing. What we must recognize is, that the time for the meeting is almost upon us, and the only question pertinent is, Are those conducting the organic work equal to the undertaking? Of this there can be no doubt. This question being settled removes it from the field of discussion. The evidence from every quarter now is, that each one feels as they should the importance of sinking all personality, and of moving with a united effort for the accomplishment of the great end or purpose in view,—that is, a profitable, a successful Dental Congress.

Individuals in their rational thoughts and actions have always in view one of two objects,—either gaining something or being something. The former can only be realized by somebody having something to give, and since each expects to be a gainer by the acquisition of either knowledge, honor, pleasure, or friends, so should each and all be as earnest in the desire that every other one should be to this extent richer for the Congress having been. But this can only be by each one contributing something or being something, as well as receiving something. There is scarcely any one so humble or so obscure in our profession but that upon some subject, or in some manner, he or she can crystallize a thought or an act which will prove useful to some other one in the same industrial line. The meetings of the Congress are not to be ideals in profit because of long dissertations on any one subject, but by terse, pithy, well-condensed thoughts on a variety of subjects. The *Dental Cosmos* editorial for April strikes the key-note when it virtually says diametrically opposite views are held on many important questions or subjects: "The opportunity of having and hearing these discussed by the ablest thinkers in dentistry from all parts of the world must have a decided value in enabling us to arrive at a better and more accurate judgment upon the issue than would be possible under less favorable circumstances or by less efficient means." Every one has a degree of intelligence, of interest and of will to make the Congress a grand success, and it is only necessary that these be exercised for the good of others, leaving out of the question, for the time, the matter of profit or return to himself, though that will necessarily come, just as surely as the sunshine follows the shower, and the richer, more delightful, will it be when secured.

I have great faith in individuality when governed by its best motives, and under what other circumstances could such an opportunity be afforded for so rich a harvest,—every one to be truly himself in the spontaneous exercise of his intelligence, his wisdom, his interest?

The aim of the Congress, as already expressed in the issued circulars, is to gather together the evidences of the material, as well as the intellectual, progress and achievements of civilization. As a result of such an aggregation every department of human endeavor can be studied, and all who avail themselves of the opportunity may be benefited.

The thinkers and workers of every department will be those who constitute the World's Columbian Exposition. Is dentistry so handicapped by jealousy and factional strife that it cannot, equally with other professions, derive the much-valued and needed advantage which will be offered? I trust not. The interchange of ideas, methods, theories, and practical experiences is just as much needed in our profession as in any other department. Nor is there any occupation where greater benefit can be secured by such intercommunion than in our own specialty. Dentistry, truly regarded as an important department of the healing art, is in this country in in its infancy. What method could be adopted which would more certainly broaden our understanding and substantially strengthen our foundations than a free interchange of thought with our English, French, and German brethren?

C. N. Peirce.

